# Evaluating the association of state regulation of community health workers on adoption of standard roles, skills, and qualities by employers in select states: a mixed methods study

**DOI:** 10.1186/s12960-021-00684-y

**Published:** 2021-12-04

**Authors:** Tammie M. Jones, Alex Schulte, Suhashini Ramanathan, Meron Assefa, Srilatha Rebala, Peggy J. Maddox

**Affiliations:** Department of Health Administration and Policy, George Mason University, Fairfax, VA USA

**Keywords:** Community health worker, Workforce, State regulation, Certification, Core competencies

## Abstract

**Background:**

The occupation of community health worker (CHW) has evolved to support community member navigation of complex health and social systems. The U.S. Bureau of Labor Statistics formally recognized the occupation of community health worker (CHW) in 2009. Since then, various national and state efforts to professionalize the occupation have been undertaken. The Community Health Workers Core Consensus (C3) project released a set of CHW roles and competency recommendations meant to provide evidence-based standards for CHW roles across work settings. Some states have adopted the recommendations; however, there are a variety of approaches regarding the regulation of the occupation. As of 2020, 19 U.S. states have implemented voluntary statewide CHW certification programs. The purpose of this study was to explore the relationship between state regulation of CHWs and adoption of standard roles, skills, and qualities by employers in select states.

**Methods:**

This mixed methods study used purposive sampling of job ads for CHWs posted by employers from 2017 to 2020 in select states. Natural language processing was used to extract content from job ads and preprocess the data for statistical analysis. ANOVA, chi-square analysis, and MANOVA was used to test hypotheses related to the relationship between state regulation of CHWs and differences in skills, roles, and qualities employers seek based on seniority of state regulatory processes and employer types.

**Results:**

The mean job ads with nationally identified roles, skills, and qualities varies significantly by state policy type (*F*(2, 4801) = 26.21) and by employer type (*F*(4, 4799) = 69.08, *p* = 0.000).

**Conclusions:**

Employment of CHWs is increasing to provide culturally competent care, address the social determinants of health, and improve access to health and social services for members of traditionally underserved communities. Employers in states with CHW certification programs were associated with greater adoption of occupational standards set by state and professional organizations. Wide adoption of such standards may improve recognition of the CHW workforce as a valuable resource in addressing the needs of high-need and marginalized groups.

## Background

### History of community health workers

Community health workers (CHWs) have helped individuals and groups navigate health and social systems for decades. Individuals employed in the CHW roles provide a range of services, including outreach, community education, informal counseling, social support, and advocacy [1]. The CHW workforce is comprised of individuals from diverse race and ethnic groups, with approximately 65% identifying as Black or Latinx, 23% as white, and 10% as Native American. CHW is considered an umbrella term that encompasses a variety of roles and job titles for unlicensed public health workers whose education and experience varies from lay worker (individuals with no formal education) to individuals with some form of secondary education [2–4]. Titles often reflect the variety of services provided, including peer specialists, doula, peer support specialist, peer health educator, and promotor de salud [2, 3].

Efforts have evolved recently to professionalize the occupation. The U.S. Department of Labor (DOL) formally recognized CHWs as a distinct occupation by creating a standard occupational classification in 2009 [1]. Other national and state level efforts to develop core competencies, establish standards for training and certification programs, and improve financing mechanisms have also emerged. The American Public Health Association defines CHWs as “frontline public health workers who are trusted members of and/or have an unusually close understanding of the community served. A trusting relationship is a core assumption that supports CHWs serving effectively as liaisons or intermediaries between health/social services and the community to facilitate individual and group access to services, as well as improve the quality and cultural competence of CHW services.” The Community Health Worker Core Consensus (C3) Project defined roles and competencies (grouped as skills and qualities) are meant to serve as standards for state CHW occupational certification and training programs. Some states have adopted the recommendations; however, there remains wide variation in states’ approaches toward regulating the occupation and adoption of the role definitions and competencies by employers.

The purpose of this study was to determine whether there is a relationship between state occupational regulation (such as from state certifications) and employers use of C3 recommended roles, skills, and qualities.

### Growth of the CHW workforce

The Bureau of Labor Statistics projects the occupational outlook for CHWs to grow 13 percent nationally from 2019 to 2029 [5]. As of May 2020, the DOL reported that there were about 59,000 CHWs in the United States [6]. This number is likely under-reported due to the range of titles that CHWs go by and given some CHWs serve as unpaid volunteers. Other sources estimate the number of CHWs in the United States to be closer to 100,000 [7].

Several key policy changes have contributed to the growth in the CHW workforce. In 2010, the Affordable Care Act specifically listed CHWs as health professionals who function as members of health care teams and mandated additional navigation and coordination support, increasing the opportunity for CHWs. In addition, changes to federal Medicaid rules in 2013 opened the door for potential reimbursement for preventive services offered by CHWs [1]. Some examples of funding programs that states have implemented include Medicaid 1115 waivers, state Managed Care Contracts, and/or State Plan Amendments (SPAs) to financially support the work of CHWs. These funding mechanisms have allowed state Medicaid offices to change how they organize, pay for, and incentivize health plans and providers that serve low-income or vulnerable patient populations [8]. In recent years, the CDC supported CHW programs through the State Public Health Actions to Prevent and Control Diabetes, Heart Disease, Obesity and Associated Risk Factors and Promote School Health grant [9] and more recently through COVID-19 Prevention and Control funding [10].

National leaders have called for increased involvement of CHWs in the healthcare system, both as part of the COVID response and as a longer term strategy to build a strong public health foundation [11]. Health plans are employing CHWs to address high costs from frequent-flyers or super-utilizers, and self-insured employers are employing CHWs for their health promotion and prevention programs to keep people healthy and on the job. Hospitals and health systems are using CHWs for proactive community engagement and post-acute care coordination to reduce 30-day readmissions and uncompensated care costs. Clinics and other outpatient offices are using CHWs to manage high-need chronic care patients and improve provider productivity [12].

Across these settings, employers report integrating CHWs into multidisciplinary teams to address pressing public health and healthcare needs, including improving service access and quality while reducing cost due to unnecessary utilization of services. Current literature is replete with articles citing the value and/or impact of CHWs on improving chronic disease outcomes [13–17], increasing access to health care services [18, 19], reducing unnecessary hospitalizations [20–24], and overall adding value to healthcare systems [25, 26].

Community knowledge, shared life experiences, and relationships built on trust enable CHWs to address root causes of health issues in ways traditional health systems often fall short (due to lack of time, cultural competence, and/or community linkages) [27]. Continued growth in demand for CHWs will be driven by business models demonstrating success, policies that influence the evolution and expansion of CHW roles, standardization of the skills needed, and improved quality of jobs and career paths available to CHWs.

### CHW regulation

There has been a shift towards increased state regulation, specifically certification, of the CHW occupation to ensure standardization and quality of the role, due to growth in employment and new types of organizations employing CHWs [1]. As of 2020, 19 U.S. states have implemented voluntary statewide CHW certification programs. Professionals who serve in a variety of roles or job titles may elect to pursue CHW certification [28]. Certification is seen as a mechanism to build a workforce with a common set of core skills, abilities, knowledge base, and training, signaling competency to employers, payers, and other members of health care teams [29].

CHW certification is a specific form of credentialing related to recognizing an agreed-upon set of occupational standards, with certification itself often being voluntary. Broader occupational credentialing options also include licensure, registration, and permitting. To our knowledge, no states require licensure for CHWs [30, 31].

Certification offers of number of benefits to CHWs and employers, including standardization and legitimization of the role of CHWs, conferring opportunities for educational and career advancement, improving employment stability, assuring standard competencies for individuals practicing as CHWs, and increasing funding for services provided by CHWs [2, 32]. Many states institute CHW certification programs as a means to establish a reliable indicator for CHW qualifications, formally recognize the profession, and meet reimbursement requirements [30]. CHW certification may also lead to more successful CHW programs in healthcare systems [33, 34].

In spite of the benefits that come with certification, many feel that the trusted relationship between CHWs and their ethnic, geographic, or marginalized communities may be threatened by increased regulation and the possibility of “medicalization” of the field. To stem this concern, states allowed “grandfathering” of those who have practiced as a CHW prior to healthcare systems and insurers incorporating CHWs into their systems. This practice provides an opportunity to retain well regarded and experienced CHWs in the workforce while introducing a larger number of individuals who possess knowledge and skills aligned with recognized standards. For opponents of regulatory conformance there remains a concern that while certification may contribute to professionalization and establish a standard of quality for selected skills, it may also create barriers to community aligned practice and entry into the workforce due to the cost of certification [35].

### State differences in CHW certification

There are no national certification standards; however, C3 offers a single set of roles, skills, and qualities endorsed by major national stakeholder groups. The C3 standards are intended to be useful to states in developing regulatory requirements for CHW practice and/or qualification for practice. Despite the efforts of CHW advocates, such as C3, wide variation in how state regulation and certification programs are carried out remains [36]. Key differences in CHW programs across states are characterized by the maturity of the program, type of certifying organization (i.e., public or private), the entity certified (CHWs, CHW training programs and/or CHW training program faculty), and the cost of certification. The typical requirements for CHW certification focus on specific competencies (e.g., roles, skills, and qualities) that individual CHWs must meet. Some differences in select state CHW programs are displayed in Table 1. These states were selected, because they show a diversity of states with mature, new and no CHW certification programs.

**Table 1:** CHW policy in select states

	Mature policy	New policy			No policy		
	Texas	Rhode Island	Virginia	Pennsylvania	Tennessee	Alabama	Wyoming
Statewide CHW certification	Yes	Yes	Yes	Yes	No	No	No
Start date	2002 [37]	2016 [38]	2018 [39]	2019 [40]	n/a	n/a	n/a
Certifying organization	TX Dept of State Health Services (public)	RI Certification Board (private)	VA Certification Board (private)	PA Certification Board (private)	n/a	n/a	n/a
Entity certified	Individuals, training programs, and/or instructors	Individuals only	Individuals only	Individuals only	n/a	n/a	n/a
Certification cost	No cost	$125	$100	$50	n/a	n/a	n/a

Employers in states with more mature CHW certification programs are expected to demonstrate increased standardization in practice competencies. Presumably standardization is reflected in reduced variation in job ads as employers recruit for a consistent workforce that meets established competencies and quality standards.

## Methods

This study of CHW employment trends and state policy changes was conducted in three phases that were comprised of qualitative interviews, analysis of job ads using Natural Language Processing (NLP), and job ad content analysis. The first phase included semi-structured interviews with nine CHW subject matter experts. The experts included individuals and representatives from a state-level CHW program, CHW certifying organization, state CHW association, Medicaid managed care CHW program director, and chair of a state level CHW council, to name a few. These experts provided valuable insights that helped shape the design of this study.

The second phase, NLP, included extracting jobs ads derived from a particular performance period and analyzing the text of job ad content. Job ad data were obtained from a labor market research software company named Chmura. The company provides proprietary labor market software, JobsEQ that collects job ad data daily using Real-Time Intelligence. A sample of job ads posted by employers in Alabama, Maryland, Rhode Island, Tennessee, Texas, Virginia, and Wyoming from 2017 to 2020 were retrieved from JobsEQ using Standard Occupation Code (SOC) 21–1094 Community Health Workers and the following titles: community health worker, peer health educator, peer specialist, peer support specialist, doula, and promotor de salud. These commonly used titles for CHWs were identified through literature review and the SME interviews. The query resulted the job title, employer, and job ad URL for each job ad.

The job ad URLs were used to generate a unique identifier for each observation and retrieve the complete job description for each ad. The observations were deduplicated using the unique identifier. Employers were categorized by type as (1) hospitals/health systems, (2) other non-hospital healthcare, (3) health department, (4) health plans, (5) community-based organizations, (6) other, and (0) unknown. The ads categorized with employer type unknown or other and those that did not include one of the CHW titles in the job title or job description were removed to reduce false positive results for CHW related job ads. The final sample included 4804 deduplicated job ads. Next, job ad text was parsed and tokenized into three-word patterns (trigrams). Then the list of roles, skills, and qualities for community health workers identified by the Community Health Worker Consensus Project [41] were lemmatized, a text pre-processing technique in which words are reduced to their root. A binary variable was created for each lemmatized key word and assigned 0 if the ad did not include the word(s) or 1 if it included the word(s). A composite variable for roles, skills, and qualities was generated from the sum of values for individual key words under each category.

In the third phase of this study, a series of hypotheses were tested using one-way analysis of variance (ANOVA), chi-square analysis (CHI2), and multivariate ANOVA (MANOVA) to examine the association between state CHW regulation (policy) and CHW roles, skills, and qualities. The first ANOVA tested differences in composite scores for skills, qualities, and roles between state policy types (i.e., no policy, new policy, mature policy). A Bonferroni post-hoc test was conducted to identify specific differences between types of policy. The second analysis used chi-square to test the association between policy type and individual key words representing CHW skills, qualities, and roles (e.g., assessment, care, and advocate). The final analysis used MANOVA to address possible threats to validity, because the data were not normally distributed and the sphericity assumption is often violated.

## Results

A total of 4804 job ads were included in this analysis. Twenty three percent of the ads were from states with no state certification program, 63% were derived from states with certification programs less than 5 years (new policy states), and approximately 14% were from a state with long standing CHW certification program (i.e., mature policy states). The majority of the job ads (63.24%) were for ‘community health workers’. An additional 1766 ads (36.76%) were identified for jobs entitled doula, peer health education, peer specialist, and peer support specialist.

The largest number of job ads were posted by community-based organizations (30.47%), followed by hospital/health systems (29.87%), and non-hospital healthcare organizations (18.61%). Table 2 displays the distribution of ads by state policy type, employer type, and occupational title. Also included in Table 2 is the frequency count of job ads identified with the standardized skills, qualities, and roles recommended for state certification programs. Across all job ads, professional (88%), health disparity (38.6%), and relationship building (35.9%) were the most commonly identified skills specified in job ads. Motivate (68.4%), self-direct (38.2%), and care (17.9%) were the most common qualities included. System navigation (35.5%), social support (16.7%), and coach (15.3%) were the top roles identified in ads.

**Table 2:** Characteristics of CHW job ads

	State regulation type							
	No policy		New policy		Mature policy		Total	
	*n*	%	*n*	%	*n*	%	*n*	%
Job ads	1112	23.15	3026	62.99	666	13.86	4804	100
*Employer type*								
Hospital/health system	288	20.07	870	60.63	277	19.30	1435	100
Non-hospital Healthcare	206	23.04	627	70.13	61	6.82	894	100
Health plan	102	40.64	109	43.43	40	15.94	251	100
Community based organization	413	28.21	879	60.04	172	11.75	1464	100
Health department	103	13.55	541	71.18	116	15.26	760	100
*Occupation titles*								
SOC 21-1094	199	94.31	12	5.69	0	0	211	100
Community Health worker	363	12.84	2012	71.17	452	15.99	2827	100
Doula	6	28.57	14	66.67	1	4.76	21	100
Peer health educator	9	12.86	61	87.14	0	0	70	100
Peer specialist	130	16.39	547	68.98	116	14.63	793	100
Peer support specialist	405	45.92	380	43.08	97	11.00	882	100
*Skills*								
Assessment	460	34.61	709	53.35	160	12.04	1329	100
Capacity building	0	0	14	63.64	8	36.36	22	100
Communication	348	18.79	1184	63.93	320	17.28	1852	100
Community	911	21.55	2700	63.86	617	14.59	4228	100
Evaluation	65	14.64	270	60.81	109	24.55	444	100
Facilitation	50	25.51	112	57.14	34	17.35	196	100
Health Disparity	3	13.04	17	73.91	3	13.04	23	100
Outreach	322	23.13	770	55.32	300	21.55	1392	100
Professional	331	19.20	1153	66.88	240	13.92	1724	100
Public health	60	15.50	215	55.56	112	28.94	387	100
Relationship building	18	29.51	36	59.02	7	11.48	61	100
Social determinant	5	2.81	152	85.39	21	11.80	178	100
Social service system	0	0	70	90.91	7	9.09	77	100
*Qualities*								
Care	835	25.40	1971	59.96	481	14.63	3287	100
Compassionate	26	20.47	91	71.65	10	7.87	127	100
Honest	28	66.67	13	30.95	1	2.38	42	100
Motivate	35	11.01	235	73.90	48	15.09	318	100
Patient	373	20.30	1172	63.80	292	15.90	1837	100
Reliable	241	27.93	537	62.22	85	9.85	863	100
Self-direct	73	31.74	139	60.43	18	7.83	230	100
*Roles*								
Advocate	330	19.38	1182	69.41	191	1.22	1703	100
Care coordination	124	27.13	252	55.14	81	17.72	457	100
Case management	168	23.27	435	60.25	119	16.48	722	100
Coach	157	19.48	567	70.35	82	10.17	806	100
Cultural	181	24.63	465	63.27	89	12.11	735	100
Direct service	10	6.33	141	89.24	7	4.43	158	100
Health education	20	4.64	299	69.37	112	25.99	431	100
Mediation	0	0	38	88.37	5	11.63	43	100
Social support	19	6.62	245	85.37	23	8.01	287	100
System navigation	1	5.26	18	94.74	0	0	19	100

The average number of jobs ads by identified roles (*F*(2, 4801) = 27.97, *p* = 0.000), skills (*F*(2, 4801) = 38.17, *p* = 0.000), and qualities (*F*(2, 4801) = 2.23, *p* = 0.006) varied significantly based on state policy type (Table 3). The Bonferroni post hoc test indicates that the mean job ads that include roles and skills are significantly different between all state policy types (*p* < 0.05).

**Table 3:** ANOVA results for state policy and composite scores for CHW roles, skills, and qualities

State policy type	Roles			Skills			Qualities		
	Mean	SD	*p* value	Mean	SD	*p* value	Mean	SD	*p* value
No policy	0.91	0.99	0.000	2.31	1.36	0.000	1.45	0.99	0.006
New policy	1.20	1.20		2.45	1.46		1.37	1.04	
Mature policy	1.06	1.13		2.91	1.45		1.40	0.94	
	*F*(2, 4801) = 27.97			*F*(2, 4801) = 38.17			*F*(2, 4801) = 2.23		

The percentage of job ads with the roles advocate, care coordination, coach, direct service, health education, mediation, social support, and system navigation are significantly different by state policy type (*p* ≤ 0.05). New and mature policy states are more likely to include these roles in job ads than states without certification programs (Table 4). The percentage of job ads with skills assessment, capacity building, communication, community, evaluation, outreach, professional, public health, social determinant, and social service system are also significantly different by state policy type (*p* ≤ 0.05).

**Table 4:** Job ad role analysis, chi-square results

	Roles																			
State Policy Type	Advocate		Care Coord		Case Mgt		Coach		Cultural		Direct service		Health education		Mediation		Social support		System Nav	
	No *n* (%)	Yes *n* (%)	No *n* (%)	Yes *n* (%)	No *n* (%)	Yes *n* (%)	No *n* (%)	Yes *n* (%)	No *n* (%)	Yes *n* (%)	No *n* (%)	Yes *n* (%)	No *n* (%)	Yes *n* (%)	No *n* (%)	Yes *n* (%)	No *n* (%)	Yes *n* (%)	No *n* (%)	Yes *n* (%)
No Policy	782 (70.32)	330 (29.68)	953 (88.49)	124 (11.51)	909 (84.40)	168 (15.60)	920 (85.42)	157 (14.58)	896 (83.19)	181 (16.81)	1,067 (99.07)	10 (2.0)	1,057 (98.14)	20 (1.86)	1,077 (100)	0 (0)	1,058 (98.24)	19 (1.76)	1,076 (99.91)	1 (0.09)
New Policy	1,844 (60.94)	1,182 (39.06)	2,723 (91.53)	252 (8.47)	2,540 (85.38)	435 (14.62)	2,408 (80.94)	567 (19.06)	2,510 (84.37)	465 (15.63)	2,834 (95.26)	141 (4.74)	2,676 (89.95)	299 (10.05)	2,937 (98.72)	38 (1.28)	2,730 (91.76)	245 (8.24)	2,957 (99.39)	18 (0.61)
Mature																				
Policy	475 (71.32)	191 (28.68)	574 (87.63)	81 (12.37)	536 (81.83)	119 (18.17)	573 (87.48)	82 (12.52)	566 (86.41)	89 (13.59)	648 (98.93)	7 (1.07)	543 (82.90)	112 (17.10)	650 (99.24)	5 (0.76)	632 (96.49)	23 (3.51)	655 (100.0)	0 (0)
*p* value	0.000	0.001	0.072	0.000	0.202	0.000	0.000	0.001	0.000	0.016										

A higher percentage of job ads in states with new or mature CHW certification programs included these skills than states without a program, with the exception of assessment, where a higher percentage of ads are found in states without a certification program) (Table 5). The percentage of job ads that included the CHW qualities care, honest, motivate, patient, reliable, and self-direct were significantly different (*p* ≤ 0.05). States with no CHW certification programs had a higher percentage of job ads that included care, honest, reliable, and self-direct. Motivate was found more often in new and mature policy states (Table 6).

**Table 5:** Job ad skills analysis chi-square results

	Skills													
State policy type	Assessment		Capacity building		Communication		Community		Evaluation		Facilitation		Health Disparity	
	No *n* (%)	Yes *n* (%)	No *n* (%)	Yes *n* (%)	No *n* (%)	Yes *n* (%)	No *n* (%)	Yes *n* (%)	No *n* (%)	Yes *n* (%)	No *n* (%)	Yes *n* (%)	No *n* (%)	Yes *n* (%)
No Policy	617 (57.29)	460 (42.71)	1,077 (100.0)	0 (0)	729 (67.69)	348 (32.31)	166 (15.41)	911 (84.59)	1,012 (93.96)	65 (6.04)	1,027 (95.36)	50 (4.64)	1,074 (99.72)	3 (0.28)
New Policy	2,266 (76.17)	709 (23.83)	2,961 (99.53)	14 (0.47)	1,791 (60.20)	1,184 (39.80)	275 (9.24)	2,700 (90.76)	2,705 (90.92)	270 (9.08)	2,863 (96.24)	112 (3.76)	2,958 (99.43)	17 (0.57)
Mature														
Policy	495 (75.57)	160 (24.43)	647 (98.78)	8 (1.22)	335 (51.15)	320 (48.85)	38 (5.80)	617 (94.20)	546 (83.36)	109 (16.64)	621 (94.81)	34 (5.19)	652 (99.54)	3 (0.46)
*p* value	0.000	0.001	0.000	0.000	0.000	0.171	0.494							

	Skills													
State policy type	Outreach		Professional		Public health		Relationship building		Social Determinant		Social Service System			
	No *n* (%)	Yes *n* (%)	No *n* (%)	Yes *n* (%)	No *n* (%)	Yes *n* (%)	No *n* (%)	Yes *n* (%)	No *n* (%)	Yes *n* (%)	No *n* (%)	Yes *n* (%)		
No Policy	755 (70.10)	322 (29.90)	746 (69.27)	311 (30.73)	1017 (94.43)	60 (5.57)	1059 (98.33)	18 (1.67)	1072 (99.54)	5 (0.46)	1077 (100)	0 (0)		
New Policy	2205 (74.12)	770 (25.88)	1822 (61.24)	1153 (38.76)	2760 (92.77)	215 (7.23)	2939 (98.79)	36 (1.21)	2823 (94.89)	152 (5.11)	2905 (97.65)	70 (2.35)		
Mature														
Policy	355 (54.20)	300 (45.80)	415 (63.37)	240 (36.64)	543 (82.90)	112 (17.10)	648 (98.93)	7 (1.07)	634 (96.79)	21 (3.21)	648 (98.93)	7 (1.07)		
*p* value	0.000	0.000	0.000	0.444	0.000	0.000

**Table 6:** Job ad qualities analysis chi-square results

	Qualities													
State policy type	Care		Compassionate		Honest		Motivate		Patient		Reliable		Self direct	
	No *n* (%)	Yes *n* (%)	No *n* (%)	Yes *n* (%)	No *n* (%)	Yes *n* (%)	No *n* (%)	Yes *n* (%)	No *n* (%)	Yes *n* (%)	No *n* (%)	Yes *n* (%)	No *n* (%)	Yes *n* (%)
No Policy	242 (22.47)	835 (77.53)	1,051 (97.59)	26 (2.41)	1,049 (97.40)	28 (2.60)	1,042 (96.75)	35 (3.25)	704 (65.37)	373 (34.63)	836 (77.62)	241 (22.38)	1,004 (93.22)	73 (6.78)
New Policy	1,004 (33.75)	1,971 (66.25)	2,884 (96.94)	91 (3.06)	2,962 (99.56)	13 (0.44)	2,740 (92.10)	235 (7.90)	1,803 (60.61)	1,172 (39.39)	2,438 (81.95)	537 (18.05)	2,836 (95.33)	139 (4.67)
Mature														
Policy	174 (26.56)	481 (73.44)	645 (98.47)	10 (1.53)	654 (99.85)	1 (0.15)	607 (92.67)	48 (7.33)	363 (55.42)	292 (44.58)	570 (87.02)	85 (12.98)	637 (97.25)	18 (2.75)
*p* value	0.000	0.073	0.000	0.000	0.000	0.000	0.001							

In the analysis to determine if C3 defined roles included in job ads varied by employer type, we observed statically significant differences between organizations and policy settings. The mean number of job ads that included roles were highest in job ads posted by non-hospital healthcare employers in states without state CHW policies (*F*(4, 1107) = 60.33, *p* = 0.000) and by hospital/health system employers in states with new policies (*F*(4, 13,021) = 18.49, *p* = 0.000) or mature CHW policies (*F*(4, 661) = 6.80, *p* = 0.000).

The mean number of job ads that included C3 defined skills were greater in community-based organizations in states without a CHW policy (*F*(4, 1107) = 40.32, *p* = 0.000), by hospital/health system employers in new policy states (*F*(4, 3021) = 11.24, *p* = 0.000), and by health plans in mature policy states (*F*(4, 661) = 5.65, *p* = 0.000). The differences in adoption or inclusion of qualities were not significantly different by employer type in states without CHW policies and in the mature policy state. The mean number of ads that included qualities were higher for hospitals/health systems in new policy states (*F*(4, 3,021) = 115.06, *p* = 0.000). The Bonferroni post-hoc tests indicated the means for roles, skills, and qualities were not significantly different between some employer types and the results varied based on state policy type. Further exploration of differences by type of employer is needed. Table 7 displays the results of the analysis of variance between by type of employer and state policy type.

**Table 7:** Anova analysis of employer type and CHW roles, skills, and qualities

Employer Type	No Policy	New Policy	Mature Policy						
	Mean	SD	*p* value	Mean	SD	*p* value	Mean	SD	*p* value
Hospital/health system	1.16	0.89	0.000	1.47	1.40	0.000	1.32	1.28	0.000
Non-hospital Healthcare	1.53	1.25		1.19	1.03		1.03	1.02	
Health plan	0.47	0.61		1.01	1.06		0.80	0.69	
Community based organization	0.49	0.70		1.00	1.01		0.84	0.93	
Health department	1.08	1.00		1.17	1.25		0.90	1.05	
	*F*(4, 1107) = 60.33			*F*(4, 3021) = 18.49			*F*(4, 661) = 6.80		
*Skills*									
Hospital/health system	1.96	1.26	0.000	2.71	1.53	0.000	2.94	1.41	0.000
Non-hospital Healthcare	2.52	1.37		2.44	1.48		2.72	1.38	
Health plan	1.24	1.28		2.44	1.96		3.90	2.27	
Community based organization	2.79	1.18		2.32	1.35		2.83	1.16	
Health Department	2.05	1.50		2.25	1.29		2.73	1.51	
	*F*(4, 1107) = 40.32			*F*(4, 3021) = 11.24			*F*(4, 661) = 5.65		
*Qualities*									
Hospital/health system	1.52	0.97	0.054	1.81	1.04	0.000	1.69	0.93	0.250
Non-hospital Healthcare	1.44	0.89		0.93	0.97		1.28	0.80	
Health Plan	0.51	0.77		1.21	1.11		0.75	0.78	
Community based organization	1.76	0.92		1.56	0.97		1.48	0.84	
Health department	0.96	0.99		0.92	0.78		0.91	0.93	
	*F*(4, 1107) = 45.67			*F*(4, 3021) = 115.06			Fy = 22.15		

MANOVA was utilized as an alternative test of validity, to address the limitations of ANOVA for susceptibility to violations of the assumption of sphericity. Using Wilk’s lamba analysis, we reject the null hypothesis that state CHW policy type (*F*(2, 4801) = 26.21), *p* = 0.000) and employer type (*F*(4, 4799) = 69.08, *p* = 0.000) have no effect on roles, skills, and qualities identified in job ads.

## Discussion

This research represents an important contribution to understanding the diffusion and adoption of occupational standards by employers. This study found state CHW policies and types of CHW employers were associated with variation in adoption of nationally defined occupational roles, skills, and qualities. The mean number of jobs that included the roles and skills were significantly higher in mature policy states. Health plans in such states may have greater standardization in how CHWs are employed and, therefore, are more likely to have job ads that incorporated the specific descriptive terms utilized in job ads. Among organizations that employ CHWs in a greater variety of roles, less standardization in the roles, skills, and qualities was evident in job ads. Although the findings were not statistically significantly, hospitals/health systems job ads were associated with a higher number of the C3 qualities. Given that CHWs and CHW programs are being leveraged by health systems for these qualities and their ability to connect with community members outside clinical healthcare settings, this finding is not surprising. Adoption of a uniform framework for regulation that specifies CHW roles, skills, and qualities needed to function across various states, organizations, and practice types may improve recognition of the CHW workforce, reduce role confusion, and ensure that the unique skillset of CHWs is utilized consistently by employers, policy makers and the public.

We acknowledge some limitations of the study. The results from this study may not be generalizable, because the study sample was derived from purposive sampling of job ads from specific states. In addition, there are important differences in how states regulate CHWs. These differences may affect employer behavior and influence the adoption of occupational standards set by state and national CHW associations. Regardless, future studies on how differences in state-level regulation may affect the professionalization of CHW occupations and influence the adoption of skills, roles, and qualities utilized by employers are needed.

## Conclusions

Unmet social needs are seen as contributing to poor health as much as lack of access to healthcare [42]. CHWs are an important service delivery resource for addressing the health of underserved communities and disparities in health, especially disparities that stem from unmet social needs. Adoption of a uniform framework for regulation that specifies CHW roles, skills, and qualities needed to function across various states and employer types may improve recognition of the workforce, reduce role confusion, and ensure that the unique skillset of CHWs are available to those with the greatest needs.

## Data Availability

The data that support the findings of this study were procured from Chmura under license for the current study, and so are not publicly available. Data may be made available from the authors upon reasonable request and with permission of Chmura.

## Abbreviations

AL: Alabama
ANOVA: Analysis of variance
C3: Community Health Workers Core Consensus Project
CDC: Centers for Disease Control and Prevention
CHI2: Chi-square
CHW: Community health worker
COVID: Coronavirus
DoL: Department of Labor
MANOVA: Multivariate analysis of variance
MCO: Managed Care Organization
NLP: Natural language processing
NLTK: Natural language toolkit
PA: Pennsylvania
RI: Rhode Island
SOC: Standard occupation code
SPA: State Plan Amendment
TN: Tennessee
TX: Texas
URL: The address of a web page
VA: Virginia
WY: Wyoming
